# Ex vivo prime editing of patient haematopoietic stem cells rescues sickle-cell disease phenotypes after engraftment in mice

**DOI:** 10.1038/s41551-023-01026-0

**Published:** 2023-04-17

**Authors:** Kelcee A. Everette, Gregory A. Newby, Rachel M. Levine, Kalin Mayberry, Yoonjeong Jang, Thiyagaraj Mayuranathan, Nikitha Nimmagadda, Erin Dempsey, Yichao Li, Senthil Velan Bhoopalan, Xiong Liu, Jessie R. Davis, Andrew T. Nelson, Peter J. Chen, Alexander A. Sousa, Yong Cheng, John F. Tisdale, Mitchell J. Weiss, Jonathan S. Yen, David R. Liu

**Affiliations:** 1 Merkin Institute of Transformative Technologies in Healthcare, Broad Institute of Harvard and MIT, Cambridge, MA USA; 2 Department of Chemistry and Chemical Biology, Harvard University, Cambridge, MA USA; 3 Howard Hughes Medical Institute, Harvard University, Cambridge, MA USA; 4 Department of Hematology, St Jude Children’s Research Hospital, Memphis, TN USA; 5 Molecular and Clinical Hematology Branch, National Heart, Lung, and Blood Institute/National Institute of Diabetes and Digestive and Kidney Diseases, National Institutes of Health, Bethesda, MD USA

**Keywords:** CRISPR-Cas9 genome editing, Sickle cell disease, Genetic engineering

## Abstract

Sickle-cell disease (SCD) is caused by an A·T-to-T·A transversion mutation in the *β*-globin gene (*HBB*). Here we show that prime editing can correct the SCD allele (*HBB*^S^) to wild type (*HBB*^A^) at frequencies of 15%–41% in haematopoietic stem and progenitor cells (HSPCs) from patients with SCD. Seventeen weeks after transplantation into immunodeficient mice, prime-edited SCD HSPCs maintained *HBB*^A^ levels and displayed engraftment frequencies, haematopoietic differentiation and lineage maturation similar to those of unedited HSPCs from healthy donors. An average of 42% of human erythroblasts and reticulocytes isolated 17 weeks after transplantation of prime-edited HSPCs from four SCD patient donors expressed *HBB*^A^, exceeding the levels predicted for therapeutic benefit. HSPC-derived erythrocytes carried less sickle haemoglobin, contained *HBB*^*A*^-derived adult haemoglobin at 28%–43% of normal levels and resisted hypoxia-induced sickling. Minimal off-target editing was detected at over 100 sites nominated experimentally via unbiased genome-wide analysis. Our findings support the feasibility of a one-time prime editing SCD treatment that corrects *HBB*^S^ to *HBB*^A^, does not require any viral or non-viral DNA template and minimizes undesired consequences of DNA double-strand breaks.

## Abstract (web summary)

Prime editing can efficiently correct the sickle-cell allele to produce wild-type haemoglobin in patient haematopoietic stem cells that engraft efficiently in mice, yielding erythrocytes resistant to hypoxia-induced sickling.

## Main

Sickle-cell disease (SCD) is an autosomal recessive disorder caused by an A·T-to-T·A mutation in the haemoglobin subunit beta (*HBB*) gene, resulting in the pathogenic sickle-cell allele (*HBB*^S^*)* encoding a Glu 6→Val (E6V) substitution. This mutation changes normal adult *β*-globin (*β*^A^) to sickle *β*-globin (*β*^S^) and replacement of normal adult haemoglobin (HbA, *α*_2_*β*_2_) with sickle haemoglobin (HbS, *α*_2_*β*^S^_2_). At low oxygen tension, HbS forms rigid polymers that cause characteristic red blood cell (RBC) shape changes and initiate a complex pathophysiology that includes haemolysis, microvascular occlusions and inflammation. Clinical manifestations include anaemia, immunodeficiency, multi-organ damage, severe acute and chronic pain, and premature death^1^.

The only FDA-approved cure for SCD is allogeneic hematopoietic stem cell transplantation. However, most patients lack ideal donors and the procedure is associated with serious toxicities, including graft-vs-host disease and graft rejection^2^. Correction of the patient’s own hematopoietic stem cells (HSCs) bypasses immune complications and eliminates the need for a tissue-compatible donor. Current strategies for therapeutic manipulation of SCD hematopoietic stem and progenitor cells (HSPCs) being examined in clinical trials include lentiviral expression of an anti-sickling *β*-like globin^3^ and the use of genome editing nucleases or base editors to activate γ-globin gene transcription for induction of foetal haemoglobin (HbF, α_2_γ_2_)^4–9^. A clinical trial using Cas9 nuclease-initiated homology-directed repair (HDR) and an adeno-associated virus type 6 (AAV6)-delivered DNA template to correct the SCD mutation^10^ was stopped due to a patient developing transfusion-dependent pancytopenia^11^. Another HDR-based strategy that uses non-viral delivery of a single-stranded oligodeoxynucleotide donor^12^ has been approved for a clinical trial. We recently reported a base editing strategy using an adenine base editor to convert the pathogenic *HBB*^S^ allele into the non-pathogenic, naturally occurring Makassar allele (*HBB*^G^)^13^. While each of these strategies has distinct advantages and disadvantages, reverting the SCD E6V substitution, which requires a T·A-to-A·T transversion, represents the most physiological approach for disease correction. However, base editors cannot convert T·A to A·T, and nuclease-mediated HbF induction or HDR-mediated correction of *HBB* requires double-stranded DNA breaks (DSBs) that cause uncontrolled mixtures of on-target loss-of-function insertion and deletion (indel) mutations^12,14^, p53 activation and chromosomal abnormalities^15–21^. Moreover, strategies that require co-delivery of the HDR template by AAV transduction^10,22^ have the potential to impair HSC engraftment^14,23^, which can result in transfusion-dependent pancytopenia^11^.

An ideal treatment for SCD would permanently revert *HBB*^S^ to the wild-type allele (*HBB*^A^) with few deleterious genomic alterations or cell state changes. Because prime editing replaces a target segment of DNA with a specified new sequence up to hundreds of base pairs in length, it enables the installation of targeted insertions, deletions and any base-to-base substitutions directly into the genome of living cells and animals without requiring DSBs^24–31^. Here we report the development of a prime editing strategy that reverts the SCD allele back to wild-type *HBB*^A^ with high on-target efficiency, low frequencies of indel by-products and minimal off-target editing. Edited SCD patient HSPCs maintained prime editing levels at 17 weeks after transplantation in mice, with an average of 42% of engrafted human erythroblasts and reticulocytes across four patient donors containing at least one wild-type *HBB*^A^ allele, indicating robust editing of hematopoietic stem cells at levels that exceed the estimated therapeutic threshold. Treated cells also showed a significant reduction in sickling when cultured in hypoxic conditions. Minimal off-target editing was detected following the analysis of over 100 experimentally identified CIRCLE-seq-nominated candidate off-target sites engaged by the prime editing system, suggesting a high degree of target DNA specificity. Taken together, our results establish an early example of therapeutic prime editing in human HSCs and demonstrate a potential strategy for a one-time autologous SCD treatment that directly corrects the sickle globin allele back to wild-type *HBB* without requiring DSBs or donor DNA templates.

## Results

### Optimizing prime editing systems for HSPCs

**Fig. 1:**
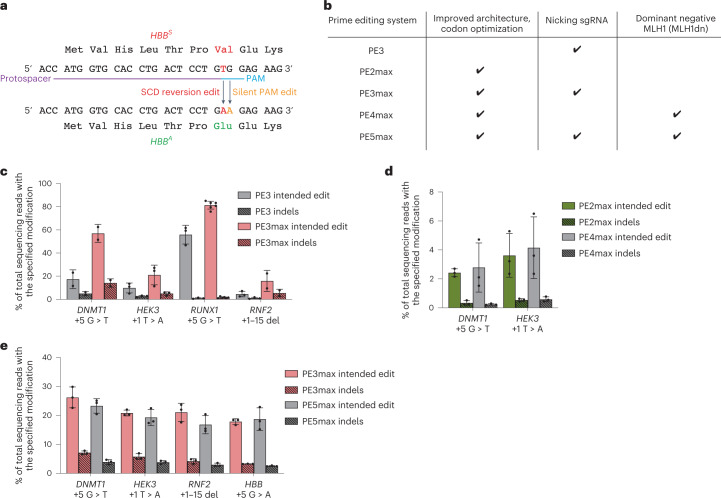
Assessment of prime editing strategies in healthy human CD34^+^ HSPCs. Cells were thawed and allowed to recover for 1 d before electroporation. Bars reflect mean ± s.d. of *n* = 2–6 independent biological replicates, with each value representing HSPCs from different donors and shown as individual dots. All intended edit values include only the desired prime editing product with no indels or other changes at the target site. Indels are shown as separate bars in each plot. **a**, PE can revert the *HBB*^S^ allele back to wild-type *HBB*^A^ by correcting the pathogenic T at position +4 (red). Including a +5 G > A PAM-disrupting edit (orange) improves editing by eliminating the NGG PAM after the edit has been made and may also help the prime editing intermediate evade MMR^25^. **b**, Chart describing the components (codon optimization, nicking sgRNA and a dominant negative form of MLH1) of each of the prime editing systems. **c**, Prime editing efficiencies at 3 d post-electroporation at various endogenous genomic loci in 5 × 10^5^ human CD34^+^ HSPCs when epegRNAs were used with the canonical PE3 system or with the PE3 system using the improved PEmax PE architecture (PE3max). **d**, Editing efficiencies for 5 × 10^5^ cells per condition for PE2max and PE4max at 3 d post-electroporation. **e**, Editing efficiencies for 5 × 10^5^ cells per condition for PE3max and PE5max at 3 d post-electroporation. While the epegRNA for *DNMT1* used in **d** and **e** encodes the same edit as in **c**, the epegRNA in **c** uses a linker designed by the pegLIT algorithm, while the linker used in **d** and **e** was designed by inspection before pegLIT was developed. Overall editing efficiency in **d** and **e** was lower at *DNMT1* compared with previous experiments, probably due to this difference. Source data

**Fig. 2:**
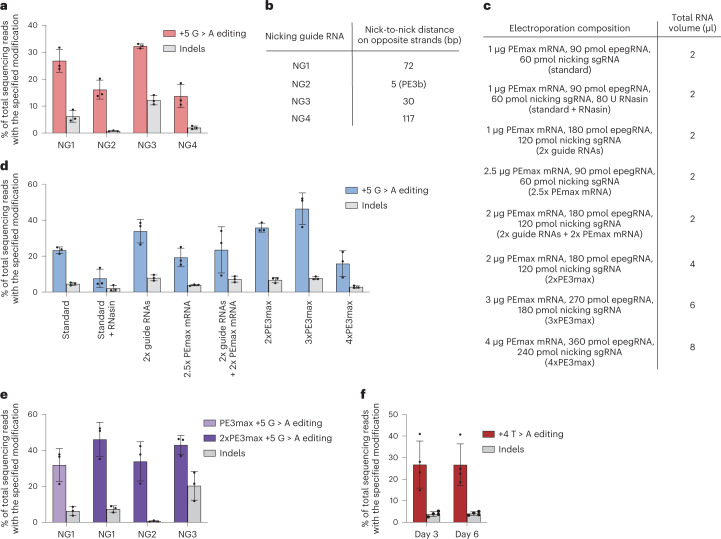
Optimization of prime editing *HBB* in human CD34^+^ HSPCs from healthy donors and from SCD patients. Healthy donor HSPCs (5 × 10^5^) and SCD patient HPSCs (4–5 × 10^6^) were electroporated. After electroporation, all healthy HSPCs and 1 × 10^5^ SCD patient HSPCs were cultured for 3 and 6 d before genomic DNA extraction. The remaining SCD patient HSPCs were cryopreserved for later mouse engraftment experiments. Bars reflect mean ± s.d. with replicate values shown as individual dots. For healthy donor HSPC editing, *n* = 3 independent biological replicates from three different donors. For SCD patient donor editing, *n* = 4 independent biological replicates from four different donors. All editing values include only the desired prime editing product with no indels or other changes at the target site. Indels are shown as separate bars in each plot. **a**, Quantification of editing efficiencies for different nicking sgRNAs targeting *HBB*. **b**, Distance between the epegRNA-induced nick and the nicking sgRNA-induced nick on the opposite strand for four nicking sgRNA candidates at *HBB*. In the PE3b strategy, nicking of the unedited strand cannot take place until after editing and ligation of the other strand is complete. **c**, Components and total combined volume of PEmax mRNA, epegRNA and nicking sgRNAs for various PE3max electroporation strategies. **d**, Editing efficiency quantification for each condition listed in **c**. **e**, Quantification of editing efficiencies using the 2xPE3max strategy with the top-performing nicking sgRNAs from **a**. **f**, Editing efficiency of reversion of the pathogenic *HBB*^S^ allele back to *HBB*^A^ in SCD patient CD34^+^ HSPCs with 2xPE3max + NG1. A total of 4–5 × 10^6^ cells were edited per donor in parallel electroporations of 5 × 10^5^ cells per replicate and pooled together for subsequent in vitro culture or cryopreserved for later injection into mice. Source data

**Extended Data Fig. 1:**
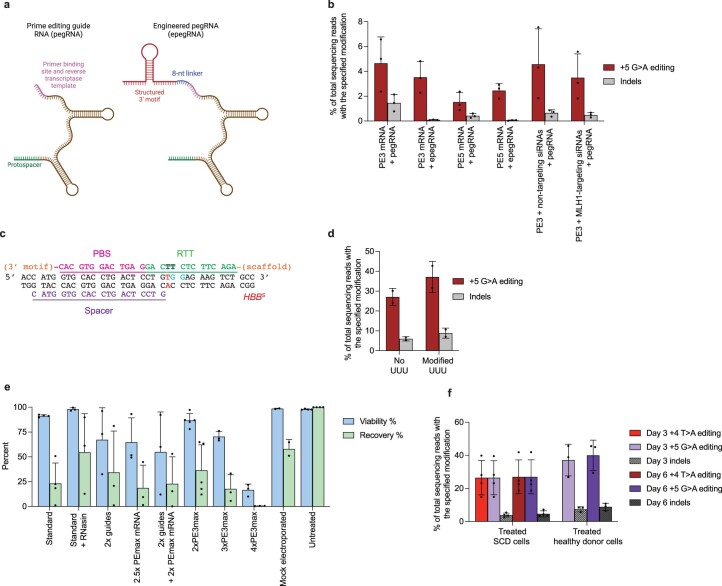
Initial PE optimization and editing outcomes at *HBB* after optimization. For all bar graphs, values represent mean+/mLSD or mean±SD, individual dots represent independent biological replicate values from separate donors, and editing efficiencies reflect only desired prime editing products with no indels or other changes at the target site. (**a**) Overview of the structural differences between pegRNAs and epegRNAs. Illustrations generated with Biorender. **(b)** Healthy human HSPCs were electroporated with PE3 or PE5 mRNA, and a pegRNA or epegRNA encoding the same +5 G>A PAM-disrupting edit at *HBB* (Supplementary Table 2). Both pegRNAs include a 5′ G in the spacer sequence and the epegRNA uses a linker designed by inspection. Separately, the PE3 mRNA + pegRNA experiment was repeated with healthy human HSPCs electroporated three days earlier with a pool of MLH1-targeting siRNAs, or with a pool of non-targeting siRNAs. 2 × 10^5^ to 5 × 10^5^ cells per condition were electroporated per condition (n = 3) with editing efficiencies measured 3 days post-electroporation. **(c)** Schematic of the PBS and RTT of the epegRNA (top line) engaged with the target *HBB*^*S*^ site (black). The SCD mutation is in red. The PAM (TGG) is the red nucleotide and the two neighboring two blue nucleotides. The epegRNA spacer is in purple and underlined, while the PBS and RTT are in pink and green, respectively. The sickle cell reversion edit (+4 T>A) and the PAM-disrupting edit (+5 G>A) encoded by the epegRNA are bolded in green. Constant regions of epegRNAs (the 3′ structured pseudoknot motif and guide RNA scaffold) are noted in orange. (**d**) 5 × 10^5^ cells per condition were electroporated with 2xPE3max with an epegRNA with or without a modified (phosphorothioate-linked) UUU trinucleotide to the 3′ end (Supplementary Table 2). Editing efficiency was measured 6 days after electroporation by high-throughput DNA sequencing. (**e**) 1 day post-electroporation, cell viability and recovery were measured using a Chemometec Nucleocounter-NC3000 for each condition (n = 2 to 5). Acridine orange and DAPI were used to stain the total cell number and dead, permeabilized cells respectively. The percent viability was calculated as the DAPI stained cells divided by the acridine orange cells within each sample. The percent recovery was normalized to the cell count of the unedited sample, which was not electroporated. All replicates with near 0% cell recovery originate from the same vial of HSPCs in which there was massive cell death upon initial thaw, before editing occurred. (**f**) Quantification of editing efficiencies for both SCD patient (+4 T>A and +5 A>G, n = 4) and healthy donor HSPCs (+5 A > G, n = 3). Source data

We previously reported the use of prime editing to correct *HBB*^S^ by plasmid transfection in HEK293T cells containing the SCD mutation, reaching up to 58% efficiency (Fig. 1a)^24^. In contrast to HEK293T cells, HSPCs are difficult to transfect with plasmid DNA but are amenable to RNA electroporation, an ex vivo delivery method that has been used in the clinic to manipulate HSPCs before transplantation^6,7,32^. We sought to identify an RNA electroporation-based prime editing strategy for HSPCs that incorporates recent prime editing advances (Figs. 1 and 2). We recently designed improved engineered pegRNAs (epegRNAs)^26^ that incorporate a 3′ structured motif to protect the reverse transcriptase template (RTT) from exonuclease degradation and an 8 nt linker that hinders potential interference between the motif and the RTT (Extended Data Fig. 1a). The linker can be designed optimally with an algorithm (pegLIT) or less effectively by visual inspection^26^. In addition, we developed PEmax, an improved prime editor (PE) architecture with optimized Cas9 and nuclear localization sequences, and codon usage^25^ (Fig. 1b). We compared editing outcomes following the electroporation of in vitro-transcribed PE and PEmax messenger RNA (mRNA) with synthetic epegRNAs in healthy human donor HSPCs to install various edits across four genomic loci—*DNMT1* (both with a fully optimized linker as in Fig. 1c and with a linker chosen by inspection as in Fig. 1d,e), HEK293T genomic site 3 (hereafter referred to as *HEK3*), *RUNX1* and *RNF2* (Fig. 1c and Supplementary Tables 1 and 2). Both PE and PEmax mRNA together with epegRNAs and nicking single-guide RNAs (sgRNAs) supported substantial prime editing efficiencies in HSPCs (up to 85%), with PEmax conferring 1.3- to 3.5-fold average increases in editing efficiency over PE (Fig. 1c). Thus, PEmax mRNA can be electroporated together with synthetic epegRNAs and nicking sgRNAs to robustly edit primary human HSPCs.

In addition to the improved PEmax architecture, our group previously reported that prime editing outcomes can be improved by inhibiting mismatch repair (MMR) using transient co-expression of MMR protein MLH1 (MLH1dn), a dominant negative MLH1 variant^25^, or by installing benign or silent mutations near the desired edit that cause the prime editing intermediate to naturally evade MMR. PE4 and PE5 systems transiently co-express MLH1dn with PE2 and PE3, respectively^25^ (Fig. 1b). Impeding MMR increases prime editing efficiencies for small substitutions, insertions and deletions and decreases the formation of indel by-products in a wide variety of cells^25,33^. To determine whether PE4max or PE5max might increase desired editing outcomes over PE2max and PE3max in primary human HSPCs, we electroporated in vitro-transcribed RNA encoding all prime editing components and MLH1dn into healthy donor cells. Surprisingly, transient MLH1 expression did not improve prime editing over PE2max or PE3max in HSPCs (Fig. 1d,e). The +5 G > T edit at *DNMT1* and the +5 G > A edit at *HBB* are silent protospacer adjacent motif (PAM) alterations that may help the prime editing intermediate natively evade MMR, without MLH1dn. However, the intermediate mismatch to install the +1 *HEK3* T > A is a known substrate for MMR^25^, and the prime editing efficiency in HSPCs of +1 HEK3 T > A also does not benefit from the addition of MLH1dn. Similarly, editing efficiencies for the +1–15 deletion at *RNF2* in primary human HSPCs also did not benefit from the addition of MLH1dn (Fig. 1d,e).

We also compared prime editing at *HBB* with PE3 versus PE5 (not ‘max’ architecture) using a pegRNA or an epegRNA in healthy human HSPCs under conditions in which prime editing was limiting and thus the benefits of MMR evasion would be most easily manifested. We observed no improvement in prime editing from MLH1dn expression (Extended Data Fig. 1b). Consistent with these results, we also pretreated primary human HSPCs with small interfering RNAs (siRNAs) targeting MLH1 and observed no benefit to PE3 prime editing outcomes at *HBB* compared with the identical treatment using non-targeting siRNAs (Extended Data Fig. 1b). These observations collectively suggest that cellular MMR may not limit the efficiency of this prime edit in HSPCs under these conditions. A recent study reports that constitutive expression of MLH1dn in a PE5max strategy shows increased editing efficiency compared with PE3max in human HSPCs edited with helper-dependent adenovirus vector^34^. Since expression from mRNA is much more transient than viral expression, we speculate that the timing of MLH1dn following mRNA electroporation in HSPCs may be less effective at enhancing prime editing outcomes than longer-lasting MLH1dn expression, or that the combination of the target edit and the adjacent silent PAM edit is sufficient to cause the prime editing intermediate to evade MMR. We therefore continued optimizations using electroporation-based prime editing systems with epegRNAs and PE3max and without MLH1dn.

### Optimizing prime editing agents to revert HBB^S^ to HBB^A^

We sought to further optimize editing outcomes at the *HBB*^S^ locus in primary human CD34^+^ cells. We designed an epegRNA that would both revert the pathogenic sickle mutation and allow optimization in healthy (homozygous *HBB*^A^) donor CD34^+^ cells by including a silent PAM edit (Extended Data Fig. 1c). We first removed the 5′ G required for efficient guide expression from plasmids and included an 8 nt linker designed by pegLIT, a computational tool that identifies non-interfering nucleotide linkers between pegRNAs and 3′ epegRNA motifs^26^. Additionally, we appended a UUU trinucleotide to the 3′ end of the epegRNA for additional protection from degradation, with each uracil harbouring a 2′-O-methyl modification and with three phosphorothioate linkages, one before each 3′ uracil nucleotide^26^. On average, epegRNAs containing this modified trinucleotide conferred a 1.4-fold increase in prime editing efficiency with a similar product purity compared to epegRNAs lacking the 3′ modified UUU (Extended Data Fig. 1d). We also included a silent PAM-disrupting edit in the epegRNA (+5 G > A), which prevents the reengagement of target DNA after prime editing and serves as a marker to assess prime editing efficiencies in healthy donor CD34^+^ cells that lack the pathogenic +4 T > A *HBB*^S^ mutation.

We electroporated the newly designed synthetic epegRNA together with in vitro-transcribed PEmax mRNA and synthetic nicking sgRNA NG1 (constituting a PE3max system) into healthy human donor HSPCs (Fig. 2a). We collected genomic DNA from treated cells (without enriching for transfected cells) at 3 d following electroporation and assessed on-target editing by high-throughput sequencing (HTS). We observed the desired precise prime editing outcome without any indels or other unwanted target site changes at an average efficiency of 27% (Fig. 2a). This observation demonstrated that synthetic epegRNAs containing a modified 3′ UUU trinucleotide support substantial prime editing efficiencies in primary human CD34^+^ cells.

To further optimize the PE3max system, we screened multiple *HBB*-nicking sgRNAs (NGs, Fig. 2a,b). We tested three additional NGs, including a PE3b nicking sgRNA that cannot nick the unedited strand until after the desired prime edit has occurred on the opposite strand. We previously established that PE3b editing strategies minimize indel by-products from prime editing by reducing the frequency of intermediates containing simultaneous nicks in both DNA strands^24^. Indel formation at this target site can eliminate *HBB* expression or create non-functional mutant proteins. Indeed, the PE3b nicking sgRNA (NG2) yielded the best ratio of desired edit to indel by-products, with 16 ± 3.5% desired editing and only 0.75 ± 0.16% indels at 3 d post-electroporation (Fig. 2a). However, desired on-target editing using NG2 was lower than our original nicking sgRNA (NG1), which resulted in 27 ± 4.3% desired editing and 6.2 ± 2.2% indels. While NG3 resulted in higher on-target editing (32 ± 0.81%), this increased editing efficiency was accompanied by higher levels of indels (12 ± 1.8%). NG4 achieved only 14 ± 4.3% editing with 2.0 ± 0.58% indels. Together, these results establish that the choice of the nicking sgRNA substantially impacts *HBB*^S^ prime editing outcomes.

### Optimizing ratios and volumes of electroporation editing reagents

Next, we identified efficiency bottlenecks in ex vivo prime editing of HSPCs and determined the optimal ratio of PEmax mRNA to synthetic epegRNA and nicking sgRNA. Keeping the guide RNAs and PEmax mRNA at 10% of the total electroporation volume following the Lonza 4D manufacturer’s recommendation, we doubled the concentration of guide RNAs, PEmax mRNA or both (Fig. 2c). Additionally, we doubled, tripled or quadrupled the total volume of all RNAs added per electroporation beyond the manufacturer’s suggestion. Finally, we tested the addition of RNasin, which has been previously reported to increase the efficiency of RNA-based electroporations by inhibiting endogenous RNase activity^35^ (Fig. 2c).

Among these variables, we found that increasing the total volume of editing reagents, but not changing the ratio of PEmax mRNA:guide RNAs, or adding RNasin, had the largest effect on prime editing outcomes. The standard volumes of PEmax mRNA and guide RNAs resulted in 23% desired editing without other target site changes. However, increasing the volume of the delivered RNAs to 2-fold higher than the amount recommended by Lonza increased on-target editing to 36% (Fig. 2d). A 3-fold increase of RNA editing reagents over the recommended volumes further increased on-target editing to 46%. However, cell viability decreased from 87% to 70% (Extended Data Fig. 1e). A 4-fold increase over the suggested volume of RNAs substantially reduced editing efficiency to 16% and greatly reduced cell viability to 17%. All other conditions tested failed to outperform the 2-fold increase of delivered RNAs. Our findings show that editing efficiency can be enhanced by optimizing the ratio between the in vitro-transcribed PEmax mRNA and the synthetic guide RNAs, and that increasing the volume of editor reagents above 20% of the total volume of electroporation harms cell viability. In light of these results, we used 20% by volume PEmax mRNA and guide RNAs for all subsequent electroporation experiments.

To determine whether 20% by volume of PE3max could improve editing with different nicking guides (NG1–3), we tested and compared each nicking sgRNA (Fig. 2e). We observed increased desired editing from using the original nicking sgRNA, NG1 (46 ± 9.5% desired editing with no other target site changes), with modest indel frequency (7.4 ± 1.8%); increased editing for NG2, the PE3b nicking sgRNA (34 ± 11%) with minimal indels (0.61 ± 0.36%); and increased editing for NG3 (43 ± 5.3%) with high indel levels (20 ± 8%). Together, these results indicate that the improvement in editing efficiency from using a 2x volume of reagents in the electroporation reaction is applicable across multiple nicking sites. Since the frequency of indel-free on-target editing (46%) was highest for NG1, we used this nicking sgRNA, the epegRNA optimized above and in vitro-transcribed PEmax mRNA as our strategy to directly revert the *HBB*^S^ allele in HSPCs from SCD patients.

### HBB^S^ correction in SCD patient HSPCs

For prime editing of patient HSPCs, we thawed cryopreserved Plerixafor-mobilized CD34^+^ cells from three SCD patient donors, or CD34^+^ cells isolated from cryopreserved bone marrow from two additional SCD patient donors and then allowed them to recover for 1 d (Supplementary Table 3). Next, we electroporated the patient cells and healthy donor HSPCs in parallel using the optimized PE3max system. We maintained 100,000 cells in culture to extract genomic DNA at days 3 and 6 and cryopreserved the remaining cells for mouse engraftment experiments. Edited CD34^+^ cells from four different SCD patient donors showed an average of 26 ± 10% desired prime editing of *HBB*^S^ to wild-type *HBB*^A^ by day 3 and 27 ± 10% editing by day 6 (Fig. 2f). Compared with these samples, CD34^+^ HSPCs from a fifth SCD patient exhibited poor editing with extensive cell aggregation at day 3 and were not carried forward into subsequent experiments.

Indel frequencies in prime-edited HSPCs remained low, averaging 3.9 ± 1.2% and 4.6 ± 1.8% on days 3 and 6 respectively, reinforcing that prime editing leads to much fewer indels and much higher desired edit-to-indel ratios (5.9–6.7) than current *HBB*^S^ correction strategies that utilize Cas9 nuclease-HDR, which have reported indel frequencies of 28–45% and edit-to-indel ratios of 0.74–1.6^12,14,24^. The most efficiently edited patient HSPCs exhibited 41% *HBB*^S^-to-*HBB*^A^ correction, with 5.2% indels at day 3, representing an edit-to-indel ratio of 7.9. While the frequency of specific indel outcomes varied by donor, no single indel outcome represented more than 0.02% of total sequencing reads for any donor sample (Supplementary Tables 4–7). Editing efficiency for the silent PAM-disrupting +5 G > A edit was nearly identical to that of the +4 T > A reversion edit for all SCD patient donors, consistent with the processive mechanism of prime editing^24^ (Extended Data Fig. 1f). Thus, our optimized prime editing system robustly reverts the *HBB*^S^ allele back to wild-type *HBB*^A^ in SCD patient HSPCs with high reversion to indel ratios.

### Transplantation of prime-edited human HSPCs into mice

**Fig. 3:**
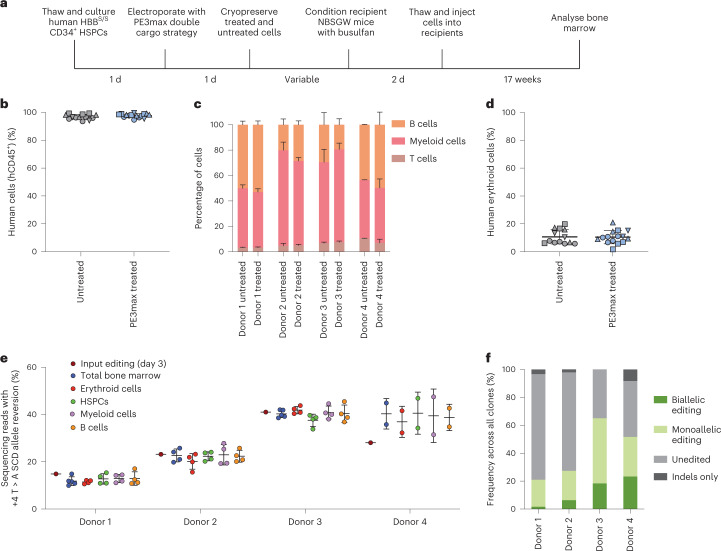
Engraftment of prime-edited SCD patient CD34^+^ HSPCs after transplantation into immunodeficient mice. We transplanted 2 × 10^5^ 2xPE3max edited HSPCs from SCD patients into the tail vein of 2–5 busulfan-treated NBSGW mice per donor. Mouse bone marrow was collected and analysed 17 weeks post-transplantation. For bar graphs, bars and error bars represent the cumulative average + s.d. (if applicable) of each lineage. For **b**, **d** and **e**, lines and error bars represent mean ± s.d., and each individual symbol represents a single mouse. **a**, Overview of engraftment experiments. **b**, Human cell engraftment in recipient bone marrow measured by percentage of human CD45^+^ cells (hCD45^+^). Each donor is coded with a unique shape: donor 1, circles; donor 2, squares; donor 3, point-up triangles; donor 4, point-down triangles. **c**, Percentages of human T cells (hCD3^+^), myeloid cells (hCD33^+^) and B cells (hCD19^+^) in the hCD45^+^ cell population in recipient bone marrow. **d**, Percentage of human erythroid precursor cells (hCD235^+^) as a percentage of human CD45^+^ and mouse CD45^−^ cells in recipient bone marrow. **e**, *HBB*^S^-to-*HBB*^A^ editing efficiency for desired editing with no indels or other undesired products at the target site across human CD34^+^ cell-derived lineages in recipient bone marrow. Each lineage was isolated using antibodies against appropriate surface markers: hCD235 for erythroid lineages, hCD34 for HSPCs, hCD33 for myeloid cells and CD19 for B cells. **f**, Average allelic editing for each of the four patient donors across 454 total BFU-E colonies derived from PE-treated CD34^+^ HSPCs. Lin^–^ cells were isolated and plated to achieve 95–120 individual colonies per donor. After 12 d in culture, colonies were picked into cell lysis buffer and desired prime editing at the *HBB* locus was measured by HTS. Colonies were categorized by whether they had a biallelic edit without indels, a monoallelic edit without indels, no desired editing or indels in at least one allele. Source data

To determine whether prime-edited HSPCs from SCD donors can repopulate bone marrow in vivo, we thawed cryopreserved prime-edited and untreated HSPCs from four SCD donors, then transplanted each via tail-vein injection into 2–5 immunodeficient NOD B6.SCID *Il2rγ*^*−/−*^*Kit*^*W41/W41*^ (NBSGW) mice, which were pretreated with low-dose busulfan at 2 d before injection to enhance donor HSPC engraftment^36^ (Fig. 3a). We collected the bone marrow of the mice for analysis 17 weeks post-injection, a time when most or all remaining human cells have been demonstrated to be derived from bone-marrow-repopulating HSCs^37^.

**Extended Data Fig. 2:**
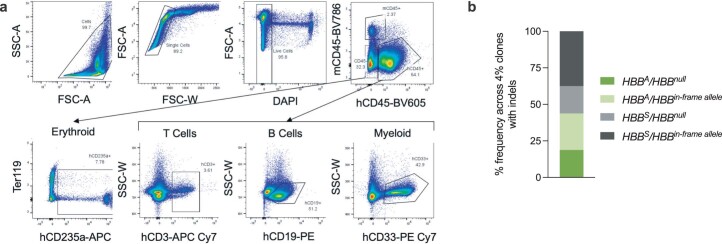
Flow cytometry analysis of human HSPC lineage populations and indel analysis of BFU-E colonies. (**a**) Representative immune-flow cytometry for T cells (hCD3^+^), B cells (hCD19^+^), myeloid cells (hCD33^+^), and erythroid cells (hCD235a^+^). SSC-A, side scatter area; SSC-W, side scatter width; FSC-A, forward scatter area; DAPI, live-dead stain. (**b**) Allelic genotypes across each of the four patient donors for the 4% of colonies (16 colonies out of 454 total BFU-E colonies) derived from PE-treated CD34^+^ HSPCs with indels as in Fig. 3f. Colonies were categorized by whether they had the intact desired reversion allele (*HBB*^*A*^), no desired editing (*HBB*^*S*^), an out-of-frame indel (*HBB*^*null*^), or an in-frame indel (*HBB*^*in-frame indel*^). Source data

The engraftment, expansion and differentiation of human HSCs can be altered by genome editing^38,39^. To investigate whether these parameters were affected by prime editing of *HBB*^S^, we used flow cytometry with human-specific antibodies to quantify human donor cells in recipient mouse bone marrow. Human cells expressing the CD45 hematopoietic antigen represented approximately 97% of all bone marrow cells in recipient mice (Fig. 3b), in which the prime-edited cells engrafted with efficiencies comparable to untreated cells, indicating that there was no engraftment impairment, in contrast with nuclease-initiated HDR methods^14^. The frequencies of human B cells (CD19^+^), myeloid cells (CD33^+^), T cells (CD3^+^) and erythroid cells (CD235a^+^) were similar in bone marrow reconstituted with prime-edited HSPCs and untreated control HSPCs (Fig. 3c,d and Supplementary Table 8). We next used lineage-specific antibodies to purify SCD patient donor mononuclear cells (CD45^+^, ‘total bone marrow’), erythroblasts (CD235a^+^), HSPCs (CD34^+^), myeloid cells (CD33^+^) and B cells (CD19^+^) (Supplementary Table 8 and Extended Data Fig. 2a) and quantified the frequency of *HBB*^S^ reversion across all lineages for each of the four donors. The prime editing frequencies of the injected cell population (15 to 41%) largely matched the levels of editing across all lineages (12 ± 0.62 to 40 ± 1.6%) recovered at 17 weeks post-transplantation (Fig. 3e). Together, these findings indicate that prime editing is retained at high frequency in bone-marrow-repopulating HSCs, which remains a challenge for some editing strategies that require DSBs^14^. Moreover, prime editing did not appear to alter HSC differentiation or maintenance of the lineages examined after bone marrow transplantation.

Of note, one donor achieved higher editing levels in all human cell populations collected from mouse bone marrow compared with input editing levels at day 3 (donor 4, Fig. 3e). This engrafted donor was the only one in which HSPCs were isolated from cryopreserved bone marrow, while all HSPC populations for all other engrafted donors were collected from Plerixafor-mobilized blood. It is tempting to speculate that the propensity of HSCs for prime editing may vary according to the HSPC source. Although the editing frequencies of repopulating cells were higher than that of input cells for this donor, all donor-derived lineages present in recipient bone marrow exhibited similar editing frequencies (Fig. 3e), consistent with our findings that PE-mediated conversion of *HBB*^S^ to *HBB*^A^ in bone-marrow-repopulating HSCs can be as efficient as that in the bulk HSPC population and that prime editing does not impact HSC lineage outcomes.

Next, we determined clonal editing outcomes among engrafted cells in mice at 17 weeks post-transplantation. We isolated human HSPCs (CD34^+^) and CD235a^+^ cells from the bone marrow of PE3max-treated or untreated mice via magnetic-activated cell sorting and seeded them into semi-solid methylcellulose medium to generate clonal burst-forming unit-erythroid (BFU-E) colonies. We isolated 454 colonies distributed approximately equally from mice transplanted with four different HSPC donors. From the different donors, we observed 21–65% of clones harbouring the precise reversion edit in one (19–47%) or both (2–23%) *HBB*^S^ alleles without any target site indels, while 35–76% were unedited (Fig. 3f). The remaining 0–8% of clones had indels in at least one allele, with 44% of those colonies also containing an intact allele with the desired edit (Extended Data Fig. 2b). These clonal editing outcomes reveal that PE3max-treated cells can be corrected at a level that substantially exceeds the 20% of corrected cells thought to be therapeutic in SCD patients^40,41^. Overall, our results demonstrate that prime-edited cells support hematopoietic repopulation and sustain predicted therapeutic levels of editing in long-term HSC populations.

### Prime editing corrects SCD characteristics in RBCs from transplanted human HSCs

**Fig. 4:**
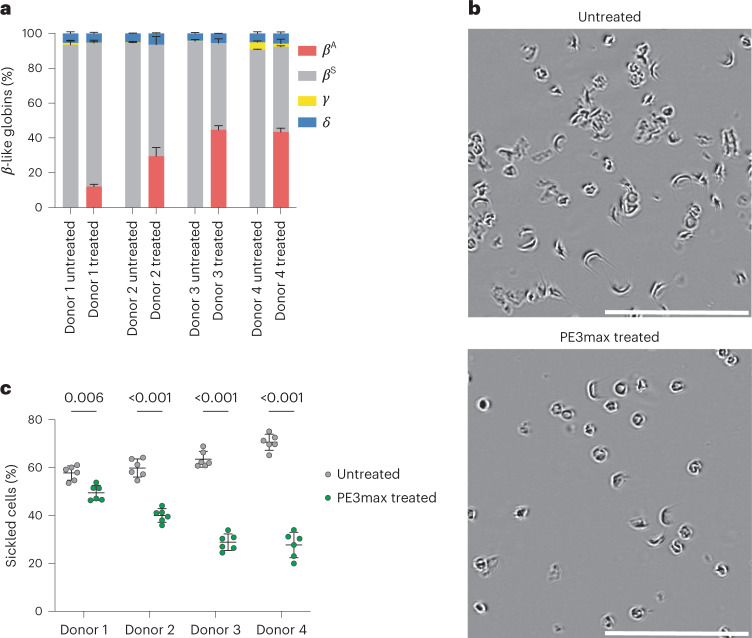
Phenotypic rescue of SCD characteristics in ex vivo-differentiated PE3max-treated human erythroid progeny from HSPCs transplanted into immunodeficient mice. **a**, Percentage of *β*-like globins measured by ion exchange HPLC in CD235a^+^ cells from human SCD patient cells at 17 weeks post-transplantation. Bars and error bars represent cumulative averages + s.d. of each protein across 2–5 mice per donor. **b**, Representative phase-contrast images of reticulocytes derived from 2xPE3max-treated or untreated SCD HSPCs incubated for 24 h with 2% oxygen. Scale bars, 100 µM. **c**, Quantification of sickled reticulocytes from images as in **b** from over 400 randomly selected cells per image. Cells were counted by a blinded observer for all conditions. Lines with error bars represent mean ± s.d., with each dot representing the percentage of sickled cells in one image from the specified donor. Significance was determined with one-sided multiple-paired *t*-tests correcting for multiple comparisons using the Holm-Šídák correction method. All *P* values are indicated. Source data

**Extended Data Fig. 3:**
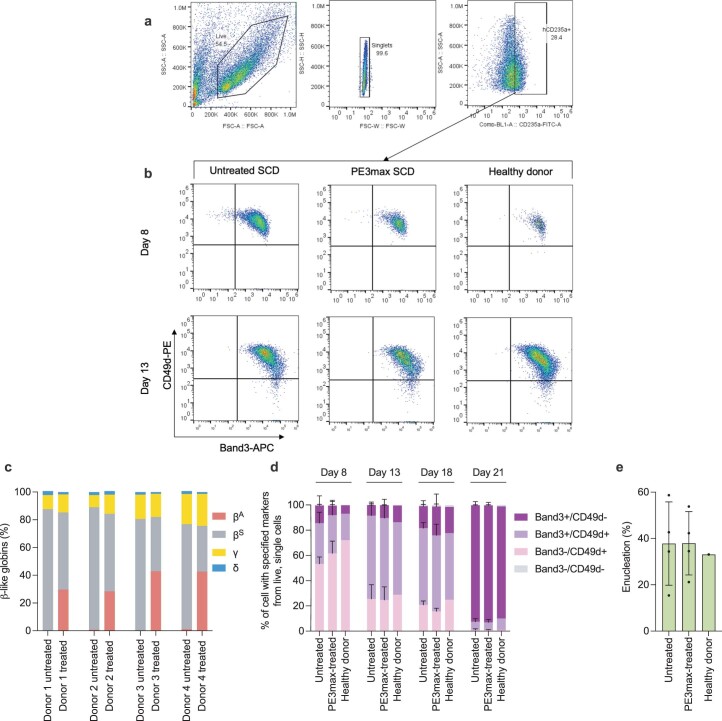
*In vitro* erythroid differentiation of SCD CD34^+^ HSPCs. (**a**) Representative immuno-flow cytometry for erythroid maturation markers at days 8 and 13 in culture for *in vitro* differentiation. Gating strategy to identify single cells hCD235a^+^ cells. (**b**) Gating strategy to track progression of erythroid differentiation based on hCD49d and Band3 expression in hCD235a^+^ cells^8,65^. SSC-A, side scatter area; SSC-W, side scatter width; FSC-A, forward scatter area. (**c**) Percentage of β-like globins measured by ion exchange HPLC in differentiated reticulocytes from human SCD patient cells. 5 × 10^4^ cells were differentiated per donor, per condition. Bars represent cumulative averages of each protein. (**d**) Quantification of terminally differentiating HSPCs *in vitro* based on Band3 and CD49d at differentiation days 8, 13, 18, and 21^8,65^ as in (b). Bars represent cumulative averages with error bars reflecting SD. (**e**) Percentage of enucleated differentiated erythrocytes at day 21. Bars represent mean±SD for n = 1–4 biological replicates from different donors, with individual dots representing values for each replicate. Source data

To determine the phenotypic impact of prime editing-mediated reversion of the sickle-cell mutation, we isolated CD235a^+^ cells from the bone marrow of transplanted mice and quantified the relative fractions of *β*-like globin proteins by high-performance liquid chromatography (HPLC). We observed a decrease in HbS and an increase in HbA that were proportional to the frequency of editing observed in total bone marrow and other lineages (Fig. 4a). Three of the four donors were edited with over 20% efficiency, with 30–45% HbA in bone-marrow-derived CD235a^+^ cells. We observed a similar result in SCD patient HSPCs differentiated toward erythroid cells in vitro (Extended Data Fig. 3a,b), with HbA production ranging from 28 to 43% (Extended Data Fig. 3c). On average, the maturation stages of erythroid precursors, including anucleate reticulocytes, were similar between in vitro-differentiated prime-edited SCD HSPCs and unedited healthy donor HSPCs (Extended Data Fig. 3d,e). These results reveal that direct reversion of *HBB*^S^ with the optimized prime editing strategy rescued HbA production, proportionately reduced pathogenic HbS levels and did not alter erythroid maturation.

The hallmark phenotype of SCD is sickling of RBCs under hypoxic conditions. To determine whether prime editing of SCD patient HSPCs to revert *HBB*^S^ to wild-type *HBB*^A^ reduces sickling in hypoxic conditions, we incubated purified reticulocytes from mice 17 weeks after transplantation and incubated them in 2% O_2_. All reticulocytes from mice receiving prime-edited cells showed a substantial reduction in sickling from an average of 63% sickled cells in untreated controls to 37% sickled cells in cells derived from prime-edited mice (Fig. 4b,c). The reduction of sickling was approximately proportional to the level of HbA in edited cells. These data together establish that prime editing can durably modify repopulating human HSCs, resulting in the production of HbA-expressing reticulocytes that resist hypoxia-induced sickling.

### Genome-wide off-target editing analysis

Studies from multiple groups have reported that prime editing causes substantially lower levels of off-target editing compared with other CRISPR gene editing methods, consistent with the mechanism of prime editing, which requires three separate base pairing events between a target DNA strand and either the pegRNA or the pegRNA-derived flap, each of which provides an opportunity to reject an off-target sequence without modification^24,28,42–50^. We assessed off-target prime editing outcomes from our *HBB*^S^ correction strategy in CD34^+^ cells. We used the experimental genome-wide off-target identification method CIRCLE-seq^51^ to nominate potential off-target loci engaged by the Cas9 domain and guide RNAs used in the prime editing experiments above, then measured off-target editing by HTS of the nominated sites in prime-edited HSPCs from SCD patient donors. Since Cas9 nuclease activity when complexed with epegRNAs is modestly decreased compared with Cas9 with the corresponding sgRNA^26^, we performed CIRCLE-seq using the optimized epegRNA, using an sgRNA surrogate containing the identical protospacer, or using the NG1 nicking sgRNA.

CIRCLE-seq nominated 515 off-target sites when using the epegRNA, 437 sites when using an sgRNA with the epegRNA spacer and 281 sites when using the nicking sgRNA (Supplementary Tables 9–11). Because a large fraction of sites nominated by CIRCLE-seq are false positives^51^, the remaining 78 loci nominated when using the epegRNA but not when using the surrogate sgRNA are probably false positives. We used RNase H-dependent amplification and sequencing (rhAmpSeq) to perform multiplex-targeted DNA sequencing of the top 50 sites for each of these three categories, excluding the on-target sites and sites not amenable to pooling with the other loci (Supplementary Table 12). Only 13 of the top 50 sites nominated using the surrogate sgRNA version of the epegRNA were not also nominated in the top 50 sites using the epegRNA. All 13 were within the top 173 sites for the epegRNA. In total, from the epegRNA and surrogate sgRNA CIRCLE-seq results, we examined in depth 63 nominated off-target candidates, including the top 50 sites from both lists.

**Fig. 5:**
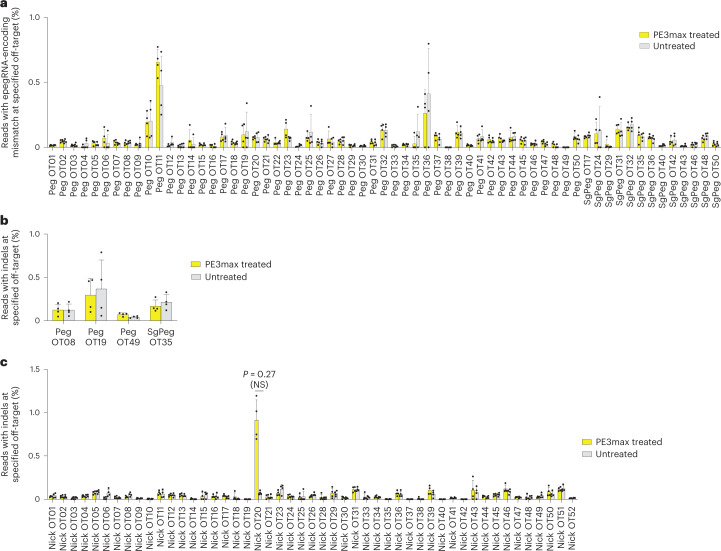
Off-target editing in prime-edited SCD patient CD34^+^ HSPCs. Bars reflect mean ± s.d. of *n* = 4 independent biological replicates from different donors, with replicate values shown as individual dots. Significance for both epegRNA and nicking sgRNA off-target editing was determined with one-sided multiple-paired *t*-tests correcting for multiple comparisons using the Holm-Šídák correction method. For all sites, the difference between PE3max-treated samples and untreated samples was not statistically significant (adjusted *P* > 0.15). **a**, rhAmpSeq quantification of the first epegRNA-encoded mismatch at CIRCLE-seq-nominated off-target loci in SCD patient HSPCs. Graph also includes epegRNA OT49 which had to be analysed separately with HTS since the primers for the locus were not amenable to pooled rhAmpSeq analysis. **b**, rhAmpSeq quantification of indels at epegRNA-nominated off-target loci nominated by CIRCLE-seq in SCD patient HSPCs. Sites for which average indel formation was >0.04% in prime-edited cells are shown. **c**, rhAmpSeq quantification of indels at nicking sgRNA off-target loci nominated by CIRCLE-seq in SCD patient HSPCs. Nick OT32 was not amenable to rhAmpSeq analysis or to PCR amplification and therefore could not be analysed. Nick OT22 was the on-target NG1 site. Source data

**Extended Data Fig. 4:**
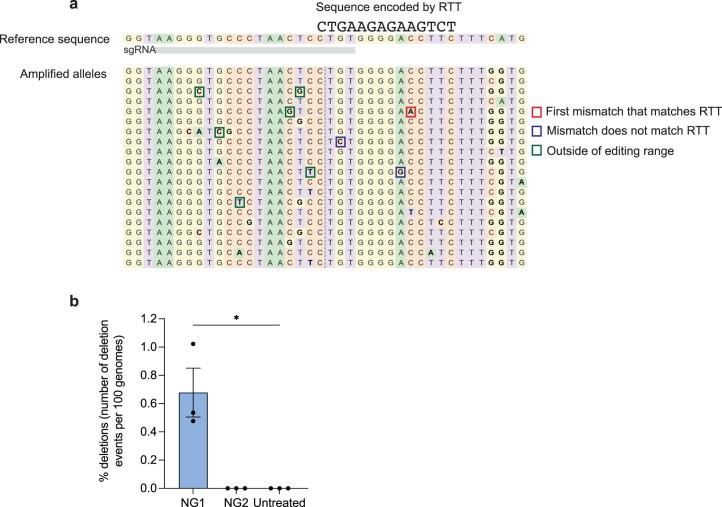
Identifying off-target editing and quantifying a deletion between *HBB* and an off-target site in *HBD*. **(a)** Shown is an example of how epegRNA-encoded off-target prime editing was identified. Since DNA sequencing errors and cellular genomic heterogeneity (observed in both edited cells and untreated cells) were the sources of the vast majority of DNA sequence differences between samples and the reference sequence, we identified potential off-target prime editing by counting sequences that contain the first mismatch encoded by the epegRNA RTT as putative epegRNA-encoded off-target prime edits. In addition to epegRNA-encoded off-target editing, nick-induced indels near sites targeted by the epegRNA or nicking sgRNAs were also separately analyzed (see Fig. 5). **(b)** Assessing the frequency of a 7.4-kb deletion between the on-target site and Nick OT20 via ddPCR. Bars represent mean±SD for n = 3 individual biological replicates from three healthy donors, with individual dots representing values for each replicate. Significance determined by a one-tailed paired t-test. *p = 0.03. Source data

To assess off-target editing at CIRCLE-seq-detected sites of engagement by Cas9·epegRNA or Cas9·surrogate sgRNA complexes, we quantified the mutation frequency at the position of the first nucleotide change that would be introduced by the epegRNA RTT at each off-target site (Extended Data Fig. 4a). This position is the most likely nucleotide to be modified during prime editing^24^. Additionally, we quantified the indel frequency at the nominated epegRNA sites. One nominated off-target site, pegOT49, was not compatible with rhAmpSeq amplification and was analysed separately. We detected no epegRNA-dependent off-target prime editing in treated cells compared with untreated controls at any of the 63 CIRCLE-seq-nominated sites (Fig. 5a,b). This high degree of DNA specificity is consistent with previous reports of low off-target prime editing and probably arises from the three distinct DNA hybridization events that must take place to result in productive prime editing^24,28,42–50^.

To determine off-target editing at CIRCLE-seq-nominated off-target sites for the nicking sgRNA, we attempted to quantify the frequency of indels at the top 50 sites. One nominated off-target site, Nick OT32, was not compatible with rhAmpSeq or PCR amplification and could not be analysed. Among the remaining 49 loci, the highest observed level of any editing (averaging 0.91%) was at Nick OT20, a genomic site that contains the identical protospacer targeted by NG1. This level of editing was not statistically significant (*P* = 0.27) in treated cells compared to untreated controls when analysed using one-sided paired multiple comparison *t*-tests correcting for multiple comparisons using the Holm-Šídák method (Fig. 5c). Nick OT20 is found in *HBD*, a lowly expressed *β*-globin gene located 7.4 kb from *HBB* on chromosome 11. We used droplet digital PCR (ddPCR) to assess the potential deletion between the on-target locus and Nick OT20. Less than 1% of genomes isolated from healthy donor HSPCs treated with 2xPE3max + NG1 contained this deletion (Extended Data Fig. 4b). This deletion was not detected when NG1 nicking sgRNA was replaced with the PE3b nicking sgRNA (NG2), consistent with the PE3b strategy of avoiding the presence of simultaneous nicks on opposite DNA strands^24^.

Collectively, the analysis of prime-edited HSPCs at a total of 112 CIRCLE-seq-nominated candidate off-target sites engaged by Cas9 complexed with the *HBB*^S^-targeting epegRNA or a corresponding sgRNA detected minimal off-target editing. Off-target indel formation for Cas9 nuclease complexed with NG1 nicking sgRNA also resulted in minimal indel formation. Although it was not statistically validated as a bona fide off-target locus, the nominated Nick OT20 site contains the same NG1 protospacer sequence that occurs within the *HBD* gene encoding haemoglobin delta, which makes up only 3% of adult globin content^52^; we also detected a rare (<1%) deletion of the intervening DNA between the on-target locus and the Nick OT20 site. The PE3b nicking sgRNA has a spacer sequence that does not match any sequence in *HBD* or any other region of human genome sequence hg38, and no deletion between *HBB* and Nick OT20 was detected in cells prime edited using the PE3b nicking sgRNA. Overall, these findings suggest that the prime editing strategy used to correct *HBB*^S^ causes minimal off-target edits in the human genome.

## Discussion

Advancements in genome editing technologies have provided many options for treating SCD. While the best strategy has not yet been determined and multiple strategies may offer clinical benefit, reverting the SCD allele back to wild type is the most physiological approach. Correction strategies using nuclease-initiated HDR face several challenges including donor DNA template delivery, perturbation of engraftment potential^14,23^ and low ratios of desired editing to indel by-products^12,14^. We describe a prime editing strategy that directly corrects the SCD allele to wild-type *HBB*^A^ without requiring double-strand DNA breaks, viral transduction or any donor DNA templates. Following extensive optimization of key parameters including the choice of PE3max, the design of the pegRNA, the total volume of RNAs electroporated and the position of the nicking guide, our strategy yields up to 41% prime editing in SCD patient HSPCs by day 3, which was maintained in bone-marrow-repopulating HSCs in transplanted mice after 17 weeks. RBCs derived from repopulated HSCs after prime editing and transplantation in mice showed a reduction in HbS and sickling, and a rise in HbA proportional to on-target editing. Ratios of *HBB*^S^ reversion to indels were high, and off-target editing was minimal after investigating 112 CIRCLE-seq-nominated candidate off-target sites.

There are several autologous transplantation approaches being developed or in clinical trials towards a treatment for SCD^3–6,10,13,22^, and it is not yet known which strategy will be the safest and most effective for patients. The prime editing strategy developed in this work offers several potential advantages. It directly eliminates the pathogenic *HBB*^S^ allele and converts it back to the wild-type allele, in contrast with strategies that rely on lentiviral expression of non-sickling globin or induction of HbF.

Current strategies to correct *HBB*^S^ in SCD patient HSCs include nuclease-initiated HDR electroporated with wild-type or a high-fidelity Cas9 nuclease complexed with a chemically modified sgRNA and a donor DNA template either delivered as single-stranded oligodeoxynucleotide donor^12^ or through recombinant adeno-associated virus serotype 6 (rAAV6)-mediated delivery^14^. Compared to non-viral donor template delivery^12^, the prime editing strategy developed in this study leads to similar levels of desired correction in patient HSCs with a much higher desired edit-to-indel ratio and far fewer off-target indels. Similarly, compared with an HDR strategy that uses rAAV6-mediated donor template delivery^14^, the prime editing strategy described here results in a higher desired edit-to-indel ratio and more efficient targeting of long-term HSCs with similar levels of pre-transplantation and post-transplantation *HBB*^S^-to-*HBB*^A^ correction. Our untreated control patient HSCs engrafted at the same level as prime-edited patient HSCs. In contrast, HSCs with rAAV6-delivered donor template engrafted significantly less efficiently than mock-electroporated cells^14^, consistent with a recent report that long-term HSCs engraft more poorly following HDR editing with rAAV6-delivered donor templates^23^. These observations suggest that prime-edited HSCs engraft at higher rates, although a well-controlled study is needed to account for any confounding variables.

Another important advantage of the prime editing strategy described here is that it does not require DSBs, although low frequencies of DSBs from staggered nicks can occur. These DSB frequencies can be virtually eliminated with a PE3b strategy that prevents simultaneous nicking of opposite DNA strands. Compared with nuclease-dependent approaches, prime editing greatly reduces the likelihood of undesired DSB outcomes such as uncontrolled and uncharacterized mixtures of indels, which in *HBB* can lead to *β*-thalassaemia-like loss-of-function^12,14^. The upper limit of tolerable indel by-products at *HBB* in the context of SCD gene correction is unknown. However, HDR-based approaches with high levels of indels may cause a *β*-thalassemia-like pathophysiology in erythroid precursors, with deficiency of *β*-globin protein, accumulation of toxic free *α*-globin, maturation arrest and apoptosis^12,53,54^. A high edit-to-indel ratio as achieved with editing methods that do not require DSBs maximizes the number of precisely corrected cells administered to patients. In comparison with nuclease-dependent approaches, prime editing also reduces the likelihood of large deletions, chromosomal loss, translocations, chromothripsis and other undesired cell state changes^15–19^. Several studies have used whole-genome sequencing, whole-transcriptome sequencing and other broad analytical methods following prime editing to assess potential genome- or transcriptome-wide changes in mammalian cells and, thus far, neither changes in single-nucleotide variants, indels, telomere length, endogenous retrotransposon activity, gene expression or splicing nor any off-target RNA editing have been reported^45,46,48–50^.

An additional feature of the prime editing strategy studied here is that it does not require DNA delivery, viral transduction or drug selection to enrich edited cells. DNA delivery is required for HDR and gene therapy, but can also lead to increased toxicity, lower engraftment frequency or insertional mutagenesis^14,21,23,55–57^.

We compared CIRCLE-seq off-target site nomination using an sgRNA containing the epegRNA spacer sequence to CIRCLE-seq using the epegRNA directly. The overlap between top hits was substantial. On the basis of the overlap and the CIRCLE-seq methodology, we suggest that either guide RNA form could be used in future assessments of off-target prime editing. Of the 112 candidate off-target sites that we assessed with HTS, only one site (Nick OT20) showed off-target editing consistently above that of untreated cells. While the observed level of off-target editing at Nick OT20 (0.91%) was not statistically significant, this candidate off-target site contains an identical protospacer sequence to the nicking sgRNA.

Converting *HBB*^S^ to the benign naturally occurring *β*-globin Makassar (*HBB*^G^) variant with an adenine base editor^13,58^ also offers advantages over Cas nuclease-based approaches and occurs more efficiently with fewer indels than reverting *HBB*^S^ to *HBB*^A^ with PE3max^13,58^. However, the prime editing approach generates the natural adult *β*-globin allele and produces fewer off-target edits than previously described *HBB*^S^-to-*HBB*^G^ adenine base editing^13,58^. Cas-independent off-target editing of DNA or RNA can occur with some base editors^59–61^ but was not detected with prime editing in several studies that investigated this possibility^24,28,42–50^. Because base editing and prime editing each have unique advantages, it is not yet clear which of the two might be a more promising strategy for SCD patients.

Future studies that combine more efficient PEs with the PE3b nicking sgRNA may further increase ratios of prime editing to indels. Additionally, alternative delivery modalities may be beneficial for maintaining cell viability and reducing off-target editing. For example, compared with the delivery of genome editing agents as mRNA, ribonucleoprotein is associated with shorter expression times of Cas9 and base editors, resulting in reduced off-target editing^13,62,63^. This trend will probably hold true for PEs. Additionally, electroporation can reduce HSPC viability, requiring a higher number of starting cells to be edited. Thus, the use of engineered virus-like particles^64^ or other non-viral delivery methods that do not require electroporation may further the therapeutic potential of genome editing approaches, including prime editing, by (1) reducing the toxicity of ex vivo gene modification and (2) raising the possibility of in vivo delivery modalities that obviate the need for HSC collection and transplantation.

The ex vivo mRNA delivery method used in this study is similar to current methods used for HSC editing in clinical trials^6,7,32^. Following extensive protocol optimization that may be applicable to PE correction of other pathogenic alleles, we achieved high levels of correction of *HBB*^S^ in SCD patient cells. With a single electroporation, cells could be efficiently edited and cryopreserved. After being injected upon thawing to minimize loss of multipotency in vitro, edited cells efficiently engrafted into animal recipients with no loss of target prime editing efficiency after 17 weeks. The observed reduction in HbS levels, increase in HbA levels and reduction in sickling propensity are suggestive of exceeding the predicted levels required for therapeutic benefit in SCD patients^40,41^. These findings are among the first to establish therapeutic prime editing of HSCs, collectively suggesting that prime editing and transplanting patient HSPCs may represent a promising therapeutic strategy as a one-time autologous treatment for SCD.

## Methods

### HTS

HTS of genomic DNA extracted from human CD34^+^ cells was performed as previously described^13^. Genomic DNA was isolated from cells using lysis buffer (10 mM Tris-HCl, pH 7.5, 0.05% SDS, 25 μg ml^−1^ proteinase K (ThermoFisher)). Lysed genomic DNA was incubated at 37 °C for 1 h, followed by heat inactivation at 80 °C for 30 min. Primers for amplification of the *DNMT1*, *HEK3*, *RNF2*, *RUNX1* and *HBB* loci are provided in Supplementary Table 1. Primers include adapters for Illumina sequencing. Following Illumina barcoding, PCR products were pooled and purified by electrophoresis with a 2% agarose gel and a QIAquick Gel Extraction kit (Qiagen), eluting with 30 µl of warm water. DNA concentration was determined using a Qubit dsDNA High-Sensitivity Assay kit (ThermoFisher) and sequencing was done on an Illumina MiSeq instrument (single-end read, 280 cycles) according to manufacturer protocols. Alignment of fastq files and quantification of editing frequency were performed using CRISPResso2 in batch mode with a window width spanning at least 10 nt past each nick site.

### PE, PEmax and MLH1dn mRNA in vitro transcription

In vitro transcription of PE2 and PEmax mRNA was performed as previously described^25,26^. Briefly, the 5′ untranslated region, Kozak sequence, PE2 or PEmax open-reading frame and 3′ untranslated region were cloned into a plasmid containing an inactive T7 (dT7) promoter. The mRNA transcription template was generated via PCR with primers that correct the dT7 promoter sequence and install a poly(A) tail. The mRNA was transcribed using the T7 High-Yield RNA kit (New England Biolabs) according to manufacturer instructions, except for full substitution of N1-methylpseudouridine (Trilink) for uridine and co-transcriptional capping with CleanCap AG (Trilink). The resulting mRNA was purified via lithium chloride precipitation and resuspended in TE buffer (10 nM Tris, 1 mM EDTA, pH 8.0 at room temperature). MLH1 dominant negative mRNA (MLH1dn) was transcribed analogously.

### Synthetic epegRNA and nicking single-guide RNA generation

Synthetic epegRNAs were ordered from Integrated DNA Technologies. Each contained 2′-*O*-methyl modifications at the first and last three nucleotides and phosphorothioate linkages between the three first and last nucleotides. For all epegRNAs except when explicitly noted, the pegLIT algorithm was used as previously described^26^ to design linkers. Synthetic nicking sgRNAs were obtained from Synthego and included 2′-*O*-methyl modifications at the first and last three bases as well as phosphorothioate bonds between the first three and last two bases.

### Isolation and culture of CD34^+^ human HSPCs

Circulating G-CSF-mobilized human mononuclear cells were obtained from deidentified healthy adult donors (Fred Hutchinson Research Center). Plerixafor-mobilized CD34^+^ cells or collected bone marrow from deidentified SCD patient donors were collected according to the protocol ‘Peripheral blood stem cell collection for SCD patients’ (ClinicalTrials.gov identifier NCT03226691), which was approved by the human subject research institutional review boards at the National Institutes of Health and St Jude Children’s Research Hospital. HSPCs were maintained in stem cell culture media: X-VIVO-15 (Lonza, 04-418Q) media supplemented with 100 ng µl^−1^ human SCF (R&D systems, 255-SC/CF), 100 ng µl^−1^ human TPO (R&D systems, 288-TP/CF) and 100 ng µl^−1^ human Flt-3 ligand (R&D systems, 308-FK/CF). Cells were seeded and maintained at a density of 1–2 × 10^6^ cells per ml.

### PE electroporation of human HSPC

Electroporations were performed with the Lonza 4D Nucleofector system using the programme DS-130. All electroporations were performed in 20 µl reactions using the P3 Primary Cell X Kit S (Lonza, V4XP) with 15 μl of supplemented P3 buffer according to manufacturer instructions. For a standard PE3 electroporation, 1,000 ng of in vitro-transcribed PEmax mRNA was mixed with 90 pmol of synthetic epegRNA and 60 pmol of synthetic nicking sgRNA in 2 μl. For PE4 and PE5 electroporations, 1,000 ng of in vitro-transcribed codon-optimized MLH1dn mRNA in 0.5 μl was also used. The 0.5 μl increase in volume is unlikely to negatively affect editing outcomes. For electroporations in which either the PEmax mRNA, the guide RNAs or both had increased concentrations, we increased the concentrations without increasing the standard volume of 2 μl. For 2xPE3max, 3xPE3max and 4xPE3max electroporations, we increased the volume of the editing reagents to 4 μl, 6 μl or 8 μl while keeping the standard concentrations. We thawed cells and allowed them to recover in X-VIVO 15 cytokine-supplemented media for 1 d before electroporation. We electroporated 5 × 10^5^ to 1 × 10^6^ cells per reaction and cultured them at a density of 2 × 10^6^ cells per ml. For SCD patient HSPCs to be transplanted into NBSGW mice, we cryopreserved the cells at 1 d post-electroporation. All epegRNA and nicking sgRNA sequences can be found in Supplementary Table 2. For electroporations with siRNAs (Extended Data Fig. 1b), 1.875 μl 100 μM pooled siRNAs (Horizon, MLH1-targeting L-003906-00-0005 or non-targeting D-001810-10-05) were electroporated per condition with 1,000 ng of in vitro-transcribed PE mRNA, 90 pmol of synthetic epegRNA and 60 pmol of synthetic NG1 nicking sgRNA.

### Cryopreservation of edited HSPCs

Edited cells (1 × 10^6^ to 2 × 10^6^) were allowed to recover in cytokine-supplemented X-VIVO 15 media for 1 d before cryopreservation. Cell pellets were collected and resuspended in equal volumes of Plasma-lyte-A media (Baxter) supplemented with 25% human serum albumin (Grifols Biologicals) and pentastarch media (Preservation Solutions) supplemented with DMSO (ATCC).

### Ethical approval for studies involving mice

Mouse studies were approved by the St Jude Children’s Research Hospital Institutional Animal Care and Use Committee under Protocol 579 entitled ‘Genetic models for the study of hematopoiesis’. Mice were cared for by staff at the St Jude Children’s Research Hospital Animal Resource Center according to the Guide for the Care and Use of Laboratory Animals of the National Institutes of Health.

### Mouse experiments

No statistical test was used to predetermine sample size. All recipient mice were randomly selected for transplantation conditions by an animal facility staff member who blindly determined which mice would receive which cells. Investigators were also blinded to the conditions of identification numbers assigned to each mouse. All assays were performed before identification numbers were matched to each experimental group.

### Transplantation of gene-edited CD34^+^ HSPCs

Gene-edited CD34^+^ HSPCs were transplanted into NOD.Cg^-KitW- 41J^ Tyr^+^ Prkdc^scid^ Il2rg^tm1Wjl^/ThomJ (NBSGW) mice.

Transplantation experiments were performed as previously described^13^ with the following exception: cryopreserved cells were thawed, counted and immediately injected into recipients. All antibodies used in the study can be found in Supplementary Table 8.

### Erythroid culture

Erythroid differentiation was completed as previously described^13^.

### Colony forming assay and analysis of clonal editing outcomes

BFU-E assays were performed and analyzed as previously described^13^.

### Haemoglobin quantification

Haemoglobin was quantified via HPLC using ion exchange columns as previously described^13^.

### In vitro sickling assay

The in vitro sickling assay was performed as previously described^13^. Briefly, erythroid cells were seeded into 96-well plates with 100 µl of phase 3 erythroid differentiation medium under hypoxic conditions (2% oxygen) for 24 h. Cells were monitored for 8 h with the IncuCyte S3 Live-Cell Analysis system and images of the cells were taken at ×20 objective. A blinded researcher quantified sickling under each condition by counting over 300 cells per condition.

### CIRCLE-seq off-target editing analysis

CIRCLE-seq off-target nomination and analysis was conducted as previously described^13^. All CIRCLE-seq-nominated sites can be found in Supplementary Tables 9–11.

### Targeted off-target amplicon sequencing and analysis by rhAmpSeq

Off-target sites nominated by CIRCLE-seq were amplified from PE3max-treated or untreated HSPCs from sickle-cell donors at 3 d post-electroporation using the rhAmpSeq system (IDT). A pooled sequencing library was generated using the rhAmpSeq design tool (primers listed in Supplementary Table 12). Genomic DNA was amplified using the pooled library according to manufacturer instructions and sequenced on an Illumina MiSeq instrument (single-end read, 270 cycles).

For epegRNA off-target analysis, we quantified the percentage of mismatches that could be encoded by the epegRNA and the percentage of indels at the top 63 CIRCLE-seq-nominated loci. For nicking sgRNA off-target analysis, we quantified the percentage of indels at the top 49 CIRCLE-seq-nominated loci. The code for each analysis can be found at https://github.com/YichaoOU/PE_off_target. Both epegRNA OT49 and Nick OT32 were not compatible with the pooled rhAmpSeq analysis. EpegRNA OT49 was analysed via HTS with forward primer 5′-ACACTCTTTCCCTACACGACGCTCTTCCGATCTNNNNTGGGTGTTATGGCCATCATGA-3′ and reverse primer 5′-TGGAGTTCAGACGTGTGCTCTTCCGATCTCCTCAACAAACTGAGGCATAC-3′. Nick OT32 could not be analysed because it was not amenable to PCR amplification.

### ddPCR assay

The ddPCR assay was performed as previously described^29^ in crude genomic DNA from unedited 2xPE3max + NG1-treated or 2xPE3max + NG2-treated HSPCs from healthy donors using *ACTB* (Bio-Rad, dHsaCNS141996500) as the reference gene. The primers used to determine the abundance of the 7.4 kb deletion between *HBB* and *HBD* were 5′-GCAAAGTGAACGTGGATGC-3′ and 5′-AAACCCAAGAGTCTTCTCTGTC-3′, and the probe sequence was 5′-AGGAGACCAATAGAAACTGGGCATGTG-3′.

### Reporting summary

Further information on research design is available in the Nature Portfolio Reporting Summary linked to this article.

## Supplementary information

Reporting Summary

Supplementary Data Supplementary tables.

## Data Availability

All data supporting the results of this study are available within the paper and its Supplementary Information. High-throughput sequencing data are available from the NCBI Sequence Read Archive database (PRJNA915048). Key plasmids are available from Addgene (depositor: D.R.L.), or from the corresponding authors on request. Source data for the figures are provided with this paper.

## Source data

Source Data for Figs. 1–5 and Extended Data Figs. 1–4 Source data.

## Code availability

The code used to conduct off-target quantification and statistical analysis can be found at https://github.com/YichaoOU/PE_off_target.
