# Supplementary material for: Ex vivo prime editing of patient haematopoietic stem cells rescues sickle-cell disease phenotypes after engraftment in mice
Source: Nat Biomed Eng. 2023 Apr 17;7(5):616–28. doi: 10.1038/s41551-023-01026-0 (PMC10195679; doi:10.1038/s41551-023-01026-0)
Supplement: Supplementary file 1 — Reporting Summary [file 41551_2023_1026_MOESM1_ESM.pdf]

nature portfolio

Corresponding author(s): Jonathan S. Yen and David R. Liu

Last updated by author(s): Mar 17, 2023

Reporting Summary

Nature Portfolio wishes to improve the reproducibility of the work that we publish. This form provides structure for consistency and transparency in reporting. For further information on Nature Portfolio policies, see our Editorial Policies and the Editorial Policy Checklist.

Statistics

For all statistical analyses, confirm that the following items are present in the figure legend, table legend, main text, or Methods section.

n/a	Confirmed	
☐	☒	The exact sample size (*n*) for each experimental group/condition, given as a discrete number and unit of measurement
☐	☒	A statement on whether measurements were taken from distinct samples or whether the same sample was measured repeatedly
☐	☒	The statistical test(s) used AND whether they are one- or two-sided<br>*Only common tests should be described solely by name; describe more complex techniques in the Methods section.*
☒	☐	A description of all covariates tested
☐	☒	A description of any assumptions or corrections, such as tests of normality and adjustment for multiple comparisons
☐	☒	A full description of the statistical parameters including central tendency (e.g. means) or other basic estimates (e.g. regression coefficient) AND variation (e.g. standard deviation) or associated estimates of uncertainty (e.g. confidence intervals)
☐	☒	For null hypothesis testing, the test statistic (e.g. *F*, *t*, *r*) with confidence intervals, effect sizes, degrees of freedom and *P* value noted<br>*Give P values as exact values whenever suitable.*
☒	☐	For Bayesian analysis, information on the choice of priors and Markov chain Monte Carlo settings
☒	☐	For hierarchical and complex designs, identification of the appropriate level for tests and full reporting of outcomes
☒	☐	Estimates of effect sizes (e.g. Cohen's *d*, Pearson's *r*), indicating how they were calculated

Our web collection on statistics for biologists contains articles on many of the points above.

Software and code

Policy information about availability of computer code

Data collection	Illumina Miseq software (version 2.6.2.1) was used on the Illumina Miseq sequencer to collect the high-throughput DNA sequencing data. Droplet digital PCR (ddPCR) droplets were read using a QX200 Droplet Reader (Bio-Rad, 1864001) and QuantaSoft software (version 1.4, Bio-Rad). Cell count and viability data were collected using the ChemoMetec Nucleocounter-NC3000 and the NucleoView NC-3000 software (version 2.1.25.12).
Data analysis	Sequences were analyzed by single-end reads and analysing amplicons for the desired sequence and indels using CRISPResso2 software (version 2.2.11a, https://github.com/pinellolab/CRISPResso2). The editing frequency for each target site was calculated as the ratio between the number of aligned reads with the desired edit and without indels to the total number of aligned reads.<br>The statistical significance of the sickling reduction between 2xPE3max-edited and untreated cells was calculated with one-sided multiple-paired t-tests correcting for multiple comparisons using the Holm-Šídák correction method with Prism 9 (version 9.4.1).<br>CIRCLE-seq data analyses were performed using open-source CIRCLE-seq analysis software (version 1.1) and the default recommended parameters (https://github.com/tsailabSJ/circleseq).<br>The editing frequency at each off-target site was calculated via a custom script (link provided in Code Availability). To calculate the statistical significance of off-target editing for 2xPE3max, we applied one-sided multiple-paired t-tests correcting for multiple comparisons using the Holm-Šídák correction method with Prism 9 (version 9.4.1).<br>Droplets generated for ddPCR quantification of the 7.4-kb deletion between HBB and HBD were analysed using QuantaSoft (version 1.4). To calculate the statistical significance of the abundance of the deletion between 2xPE3max-edited and untreated cells, we applied a one-sided t-test using Prism 9 (version 9.4.1).

For manuscripts utilizing custom algorithms or software that are central to the research but not yet described in published literature, software must be made available to editors and reviewers. We strongly encourage code deposition in a community repository (e.g. GitHub). See the Nature Portfolio guidelines for submitting code & software for further information.

Data

Policy information about availability of data

All manuscripts must include a data availability statement. This statement should provide the following information, where applicable:

- Accession codes, unique identifiers, or web links for publicly available datasets
- A description of any restrictions on data availability
- For clinical datasets or third party data, please ensure that the statement adheres to our policy

All data supporting the results of this study are available within the paper and its Supplementary Information. High-throughput sequencing data is available from the NCBI Sequence Read Archive database (PRJNA915048). Source data for the figures are provided with this paper. Key plasmids are available from Addgene (depositor: David R. Liu), or from the corresponding authors on request.

Human research participants

Policy information about studies involving human research participants and Sex and Gender in Research.

Reporting on sex and gender	The study did not involve human research participants.
Population characteristics	—
Recruitment	—
Ethics oversight	—

Note that full information on the approval of the study protocol must also be provided in the manuscript.

Field-specific reporting

Please select the one below that is the best fit for your research. If you are not sure, read the appropriate sections before making your selection.

☒ Life sciences ☐ Behavioural & social sciences ☐ Ecological, evolutionary & environmental sciences

For a reference copy of the document with all sections, see nature.com/documents/nr-reporting-summary-flat.pdf

Life sciences study design

All studies must disclose on these points even when the disclosure is negative.

Sample size	No statistical methods were used to predetermine the experimental sample sizes.
Data exclusions	No data were excluded.
Replication	Biological replicates were obtained, and the nature of each replicate is described in the associated figure legend or in Methods.
Randomization	Recipient mice were randomly selected for the transplantation cohorts.
Blinding	Blinding was not used, and mice were treated only a single time each. Mice were housed, fed and handled identically.

Reporting for specific materials, systems and methods

We require information from authors about some types of materials, experimental systems and methods used in many studies. Here, indicate whether each material, system or method listed is relevant to your study. If you are not sure if a list item applies to your research, read the appropriate section before selecting a response.

Materials & experimental systems

n/a	Involved in the study	
☐	☒	Antibodies
☒	☐	Eukaryotic cell lines
☒	☐	Palaeontology and archaeology
☐	☒	Animals and other organisms
☒	☐	Clinical data
☒	☐	Dual use research of concern

Methods

n/a	Involved in the study	
☒	☐	ChIP-seq
☐	☒	Flow cytometry
☒	☐	MRI-based neuroimaging

Antibodies

Antibodies used

Anti-Human CD235a FITC, clone GA-R2 (HIR2), BD Pharmingen, catalog # 559943
Anti-Human CD49d PE, clone 9F10, BioLegend, catalog # 304304
Anti-Human Band3 APC, clone custom, New York Blood Center Gift from X. An
Anti-Mouse CD45 FITC/BV786, clone 30-F11/30-F11, BD Pharmingen/BD HorizonTM catalog #s 561088/564225
Anti-Human CD45 BV605, clone HI30, BD Horizon, catalog # 564047
Anti-Human CD33 PE-Cy7, clone P67.6, BD Biosciences, catalog # 333946
Anti-Human CD3 APC-Cy7, clone SK7 (Leu-4), BD Pharmingen, catalog # 557832
Anti-Human CD19 (Leu-12) PE/FITC, clone 4G7/HIB19, BD Biosciences/BD Pharmingen, catalog #s 349209/555412
Anti-Human CD34 Alexa Flour 700/PE, clone 581/581, BD Pharmingen/BD Pharmingen, catalog #s 561440/555822
Anti-Human CD235a APC, clone GA-R2 (HIR2), BD Pharmingen, catalog # 551336

Validation

Anti-Human CD235a FITC, clone GA-R2 (HIR2), BD Pharmingen 559943 (1:100 for FACS) Metais et al, Blood Adv, 2019
Anti-Human CD49d PE, clone 9F10, BioLegend 304304 (1:20 for FACS) Validation: Metais et al, Blood Adv, 2019
Anti-Human Band3 APC, clone custom, New York Blood Center Gift from X. An (1:100 for FACS) Validation: Metais et al, Blood Adv, 2019
Anti-Mouse CD45 FITC/BV786, clone 30-F11/30-F11, BD Pharmingen/BD Horizon 561088/564225 (1:40 for FACS) Validation: Laggase et al, Nature Med, 2020 / Metais et al, Blood Adv, 2019
Anti-Human CD45 BV605, clone HI30, BD Horizon 564047 (1:20 for FACS) Validation: Metais et al, Blood Adv, 2019
Anti-Human CD33 PE-Cy7, clone P67.6, BD Biosciences 333946 (1:20 for FACS) Validation: Metais et al, Blood Adv, 2019
Anti-Human CD3 APC-Cy7, clone SK7 (Leu-4), BD Pharmingen 557832 (1:20 for FACS) Validation: Metais et al, Blood Adv, 2019
Anti-Human CD19 (Leu-12) PE/FITC, clone 4G7/HIB19, BD Biosciences/BD Pharmingen 349209/555412 (1:20 for FACS) Validation: Metais et al, Blood Adv, 2019 / Bradbury et al, J Immunol, 1993
Anti-Human CD34 Alexa Flour 700/PE, clone 581/581, BD Pharmingen/BD Pharmingen 561440/555822 (1:20 for FACS) Validation: Egeland et al, Transplant Proc, 1993
Anti-Human CD235a APC, clone GA-R2 (HIR2), BD Pharmingen 551336 (1:20 for FACS) Validation: Metais et al, Blood Adv, 2019

Animals and other research organisms

Policy information about studies involving animals; ARRIVE guidelines recommended for reporting animal research, and Sex and Gender in Research

Laboratory animals

The following applies to all animals used in the study: animal type: mouse; genotype: NOD.Cg-KitW-41J Tyr + Prkdcscid Il2rgtm1Wjl/ThomJ "NBSGW"; sex: female; age: 6 weeks at transplantation, 23 weeks at harvest; weight: average, 23g; supplier: The Jackson Laboratory.

Wild animals

The study did not involve wild animals.

Reporting on sex

No sex-based analyses were performed, as all animals in the study were female.

Field-collected samples

The study did not involve samples collected from the field.

Ethics oversight

The St. Jude Institutional Animal Care and Use Committee approved the use of mice in the transplantation experiments. The animal studies were performed according to relevant ethical regulations.

All studies using mice were approved by the St. Jude Children's Research Hospital Institutional Animal Care and Use Committee under Protocol 579 entitled "Genetic Models for the Study of Hematopoiesis". Mice were maintained in the St. Jude Children's Research Hospital Animal Resource Center according to recommendations in the Guide for the Care and Use of Laboratory Animals of the National Institutes of Health.

Note that full information on the approval of the study protocol must also be provided in the manuscript.

Flow Cytometry

Plots

Confirm that:

☒ The axis labels state the marker and fluorochrome used (e.g. CD4-FITC).

☒ The axis scales are clearly visible. Include numbers along axes only for bottom left plot of group (a 'group' is an analysis of identical markers).

☒ All plots are contour plots with outliers or pseudocolor plots.

☒ A numerical value for number of cells or percentage (with statistics) is provided.

Methodology

Sample preparation	Bone marrow, peripheral blood, and in-vitro cultured cells were resuspended in PBS with 0.1% BSA. Cells were filtered by using a 40-µm filter before flow.
Instrument	Attune NxT Flow Cytometer, BD FACSAria III, BD LSRFortessa
Software	FACS Diva for data collection, FlowJo for data analysis.
Cell population abundance	FACS-machine cell-sorting efficiency was confirmed by flow-cytometric analysis of post-sorted cells.
Gating strategy	FSC-A/SSC-A for mononuclear cells, followed by SSC-A/SSC-W for singlets, DAPI for DAPI-live cells. Human/mouse chimerism and lineages were analysed by using:<br>anti-Mouse CD45 FITC/BV786,<br>anti-Human CD45 BV605,<br>anti-Human CD33 PE-Cy7,<br>anti-Human CD3 APC-Cy7,<br>anti-Human CD19 (Leu-12) PE/FITC,<br>anti-Human CD34 Alexa Flour 700/PE,<br>anti-Human CD235a APC.<br><br>Erythroid maturation assessments were gated by<br>anti-Human CD49d PE,<br>anti-Human Band3 APC,<br>anti-Human CD235a FITC.<br><br>Extended Data Figs. 2 and 3 provide further details.

☒ Tick this box to confirm that a figure exemplifying the gating strategy is provided in the Supplementary Information.
